# Recalcitrant rectal stricture following circumferential endoscopic mucosal resection: novel application of endoscopic stricturotomy

**DOI:** 10.1055/a-2274-5826

**Published:** 2024-03-14

**Authors:** Julia L. Gauci, Renato Medas, Clarence Kerrison, Anthony Whitfield, Francesco V. Mandarino, Nicholas G. Burgess, Michael J. Bourke

**Affiliations:** 1 8539 Gastroenterology and Hepatology, Westmead Hospital, Sydney, Australia; 2 285211 Gastroenterology, Centro Hospitalar Universitário de São João, Porto, Portugal; 3 26706 Faculty of Medicine, University of Porto, Porto, Portugal; 4 8539 Gastroenterology and Hepatology, Westmead Hospital, Westmead, Australia; 5 School of Medicine, University of Sydney, Sydney, Australia

A 69-year-old woman underwent endoscopic mucosal resection (EMR) of a fully circumferential flat, homogeneous, granular lesion, abutting the anal verge and extending proximally for 6 cm ( Fig. 1 ). Steroid enemas were prescribed for a 6-week period. Histopathology confirmed a low-grade tubulovillous adenoma. The procedure was complicated by delayed bleeding which was treated by the patient’s local surgical team with extensive thermal coagulation.

**Fig. 1:**
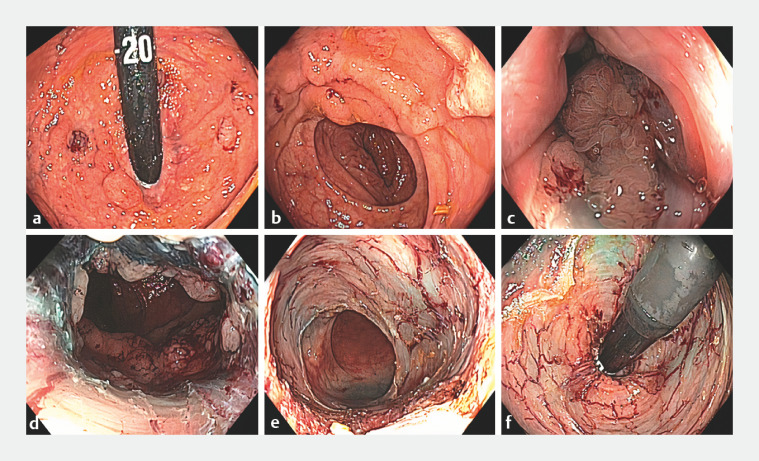
Endoscopic mucosal resection of a fully circumferential large laterally spreading rectal lesion. **a** , **b** Retroflexed and forward views. **c** Submucosal injection commenced at the dentate line. **d** Resection commenced at the distal end of the lesion, at the anal verge. **e** , **f** Complete resection with margin thermal ablation of the margin, demonstrated in forward and retroflexed views.

At 6 weeks post-EMR, the patient reported obstructive symptoms and was found to have a severe stricture, 8 mm in diameter. This was treated with sequential balloon dilatation to 12 mm at 2–4-weekly intervals. After 5 sessions, minimal progress had been made, and the patient remained symptomatic. A scarred web was noted in the 6–12 o’clock position.

Endoscopic stricturotomy was performed by creating radial incisions to the web using a 4.5-mm triangle-tip needle-knife ( Video 1 ). Subsequently, balloon dilatation to 18 mm was possible. At 12 weeks after endoscopic stricturotomy the patient remained asymptomatic. There was no recurrent or residual adenoma ( Fig. 2 ).

**Fig. 2:**
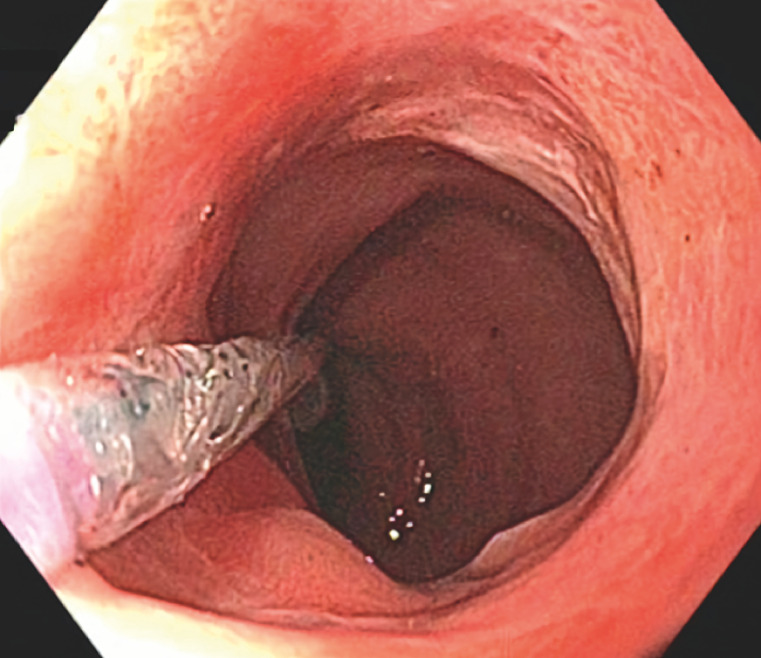
Polypectomy site 3 months after endoscopic stricturotomy and 9 months after resection, demonstrating the absence of recurrent or residual adenoma and showing a widely patent lumen with no evidence of stricture recurrence.

Successful endoscopic stricturotomy of a severe stricture, refractory to balloon dilatation, that followed circumferential endoscopic resection of a rectal large nonpedunculated colonic polyp.Video 1

Larger lesions which would have until recently required surgical resection, are now removed endoscopically in centers of excellence. Our group has previously shown that the risk of post-resection stricture formation increases with the lateral extent of the resected area 1 . A post-EMR defect involving ≥90% of luminal circumference carried a 74.2% risk of stricture formation. The median number of dilatation sessions required for severe post-endoscopic resection strictures was 3. Extensive thermal therapy to treat bleeding likely played a role in the degree of scar formation in this case.

Endoscopic stricturotomy has been utilized in the management of Crohn’s and anastomotic strictures with success 2 3 . To our knowledge, there are no published data on the management of strictures following endoscopic resection. Here we demonstrate the first case of endoscopic stricturotomy in the management of a refractory post-EMR stricture, which was safe and effective.

Endoscopy_UCTN_Code_TTT_1AQ_2AF

## Notes

Endoscopy E-Videos https://eref.thieme.de/e-videos
*E-Videos* is an open access online section of the journal *Endoscopy* , reporting on interesting cases and new techniques in gastroenterological endoscopy. All papers include a high-quality video and are published with a Creative Commons CC-BY license. Endoscopy E-Videos qualify for HINARI discounts and waivers and eligibility is automatically checked during the submission process. We grant 100% waivers to articles whose corresponding authors are based in Group A countries and 50% waivers to those who are based in Group B countries as classified by Research4Life (see: https://www.research4life.org/access/eligibility/ ). This section has its own submission website at https://mc.manuscriptcentral.com/e-videos .

## References

[1] Gupta S Vosko S Shahidi N Endoscopic resection-related colorectal strictures: risk factors, management, and long-term outcomes Endoscopy 2023 55 1010 1018 10.1055/a-2106-6494 37279786

[2] Lan N Shen B Endoscopic stricturotomy with needle knife in the treatment of strictures from inflammatory bowel disease Inflamm Bowel Dis 2017 23 502 513 10.1097/MIB.0000000000001044 28296818

[3] Dezheng L Wei L Zexian C Endoscopic stricturotomy for patients with postoperative benign anastomotic stricture for colorectal cancer Dis Colon Rectum 2022 65 590 598 34775404 10.1097/DCR.0000000000001944

